# Non-alcoholic Fatty Liver Disease (NAFLD) is associated with impairment of Health Related Quality of Life (HRQOL)

**DOI:** 10.1186/s12955-016-0420-z

**Published:** 2016-02-09

**Authors:** Pegah Golabi, Munkhzul Otgonsuren, Rebecca Cable, Sean Felix, Aaron Koenig, Mehmet Sayiner, Zobair M. Younossi

**Affiliations:** Betty and Guy Beatty Center for Integrated Research, Inova Health System, Falls Church, VA USA; Center For Liver Disease, Department of Medicine, Inova Fairfax Hospital, Falls Church, VA USA

## Abstract

**Background:**

NAFLD impacts patient reported outcomes (PROs). Our aim was to assess the impact of NAFLD on patients’ HRQOL.

**Methods:**

National Health and Nutrition Examination Survey (NHANES) 2001–2011 data were used to identify adult patients with NAFLD [Fatty Liver Index (FLI) > 60 in absence of other liver disease and excessive alcohol >20 g/day for men, >10 g/day for women]. Patients with other chronic diseases (ex. HIV, cancer, end-stage kidney disease) were excluded. Subjects without any of these conditions were healthy controls. HCV RNA (+) patients were HCV-controls. All patients completed NHANES HRQOL-4 questionnaire. Linear regression determined the association between NAFLD and HRQOL components adjusting for age, gender, race, and BMI.

**Results:**

Participants with complete data were included (*n* = 9661); 3333 NAFLD (age 51 years and BMI 34 kg/m^2^); 346 HCV+ (age 49 years; BMI 27 kg/m^2^) and 5982 healthy controls (age 48 years and BMI 26 kg/m^2^). The proportion of subjects rating their health as “fair” or “poor” in descending order were HCV controls (30 %) NAFLD (20 %) and healthy controls (10 %) (*p* < 0.001). HRQOL-4 components scores 2–4 were lowest for HCV, followed by NAFLD and then healthy controls (p-values *p* = 0.011 to < .0001). After adjustment for age, gender, race, and BMI, NAFLD patients were 18–20 % more likely to report days when their physical health wasn’t good or were unable to perform daily activities as a result (*p* < .0001).

**Conclusions:**

NAFLD causes impairment of HRQOL. As NAFLD is becoming the most important cause of CLD, its clinical and PRO impact must be assessed.

## Background

Non-alcoholic fatty liver disease (NAFLD) is an important cause of chronic liver disease worldwide [1–5]. NAFLD has been associated with cirrhosis, hepatocellular carcinoma and is currently the second indication for liver transplantation [6].

NAFLD is increasingly being diagnosed in patients with nonspecific symptoms with incidental elevation of aminotransferases [7, 8]. Besides fatigue, NAFLD patients may also experience other symptoms such as anxiety, depression, cognitive impairment, and loss of self-esteem [9]. These symptoms significantly impact patients’ well-being and health-related quality of life (HRQOL) [10]. Although the impact of chronic hepatitis C infection on HRQOL has been reported extensively, there is little published data about HRQOL assessment in patients with NAFLD. Therefore, our aim was to assess the impact of NAFLD on patients’ HRQOL as compared to HRQOL impairment in patients with CH-C and those without liver disease.

## Methods

### Study design and population

**Fig. 1:**
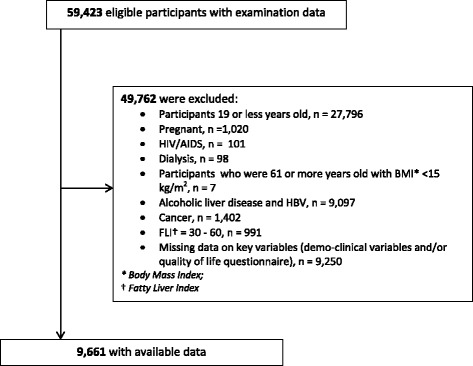
Flow chart for Eligibility, NHANES, 2001-2012

Availability of data and materials: National cross-sectional health survey data [National Health and Nutrition Examination Survey (NHANES) conducted between 2001 and 2011] were used. The Center for Disease Control and Prevention (CDC) collected the data by an interviewer-administered questionnaire conducted in participants’ homes by trained interviewers. Clinical data were obtained through the use of specially designed and equipped mobile examination centers. Participants (*n* = 49,762) were excluded if they had Alcoholic Liver Disease (ALD) (alcohol consumption of ≥ 20 g/day for men, ≥ 10 g/day for women over past 12 months), other chronic diseases such as HIV, cancer, end-stage kidney disease and/or had missing data on key variables (demographic and HRQOL), (Fig. 1- Flow chart for Eligibility, NHANES, 2001–2012)

### Sociodemographic variables

Age (years), gender, race/ethnicity (white, black, other), education (less than high school, high school, college or above), annual household income (less than $55,000 per year, $55,000 or more) were self-reported. Anthropometric measurements (height, weight) were obtained by trained staff during the medical examination.

### Definitions of chronic liver diseases

The following liver diseases were identified and included in the study: (1) *Chronic Hepatitis C diagnosis was* based on a positive Hepatitis C antibody (ELISA II analysis) or HCV RNA as detected by polymerase chain reaction; (2) *NAFLD* was determined by the Fatty Liver Index (FLI). A FLI score of 60 or more in the absence of HCV, HBV and significant alcohol consumption was NAFLD; and (3) Participants without any chronic liver diseases and a FLI score ≤ 30 were considered as Healthy Controls.

### Assessment of quality of life

Four components of a participants’ health-related quality of life (HRQOL-4) over a previous 30-day period were assessed during the scheduled visit. The four component questions were: Component 1 (C1): Rate your health status as poor, fair, good, very good, or excellent. ; (b) C2 Thinking about your physical health, which includes physical illness and injury, for how many days during the past 30 days was your physical health not good; (c) C3 Thinking about your mental health, which includes stress, depression, and problems with emotions, for how many days during the past 30 days was your mental health not good; and (d) C4 About how many days did poor physical or mental health keep him/her from doing your usual activities, such as self-care, work, or recreation.

### Data analyses

For analyses, participants were divided into two groups: fair/poor coded as “1” and good/very good/excellent were coded as “0” for component 1. For components C2–C4, we coded participants who reported no poor days (50 % of participants) as “0” and coded the others who reported any number of days >0 as “1”.

Simple logistic regression models with linearized variance estimation and weighting were used to estimate the association between self-reported quality of life questionnaire (for each component of C1–C4, separately) and liver disease while adjusting for age, gender, race, BMI, smoking, education, diabetes, and heart disease. We did not adjust household income in the final model due to a high correlation with education (Spearman rank-order rho = 0.40, *P* < .0001). Spearman rank-order correlations were estimated to examine an association between the participants’ ranking their health condition (C1) and demographic variables stratified by liver disease. SAS V9.3 was used for analyses (SAS Institute, Cary, NC). To account for the complex survey design from NHANES, SAS survey command with sampling weight, stratification and clustering variables were used except for Spearman’s rank-order correlation analyses.

## Results

### Subjects’ baseline characteristics

A total of 9661 participants with complete data were enrolled in the study (Table 1). Of these, 346 patients had HCV, 3333 had NAFLD and 5982 were considered controls. Mean age of the patients was 48.8 years (48.8 ± 0.50, *p* < .0001 in HCV, 51.3 ± 0.36, *p* < .0001 in NAFLD and 47.5 ± 0.39, *p* < .0001 in Control). NAFLD patients had significantly higher body mass index (BMI) as compared to the Controls (33.66 ± 0.14 vs 26.22 ± 0.10, *p* < .0001) and HCV patients (33.66 ± 0.14 vs 27.26 ± 0.41, *p* < .0001). Of the NAFLD cohort, 57.8 % were male and 72.4 % were White. Furthermore, metabolic syndrome components such as hyperlipidemia, hypertension, and insulin resistance were more common in the NAFLD group, as well as history of heart disease (all *p* < .001).

**Table 1:** Characteristics of Study by Liver Disease, NHANES, 2001–2012

Variables	HCV	NAFLD	Control	*P* value^a^	*P* value^b^	*P* value^c^
	(*n* = 346)	(*n* = 3333)	(*n* = 5982)			
Age (years): mean (SE)	48.86 (0.50)	51.31 (0.36)	47.50 (0.39)	0.058	<.0001	<.0001
Body Mass Index (kg/m^2^): mean (SE)	27.26 (0.41)	33.66 (0.14)	26.22 (0.10)	0.037	<.0001	<.0001
Male	228 (66.87 %)	1817 (57.79 %)	3103 (50.03 %)	<.0001	<.0001	0.014
Race,				<.0001	0.034	<.0001
White	134 (63.53 %)	1612 (72.46 %)	3221 (75.15 %)			
Black	137 (22.87 %)	684 (10.88 %)	1164 (9.50 %)			
Other	75 (13.60 %)	1037 (16.66 %)	1597 (15.35 %)			
Comorbid disease,						
Diabetes	56 (13.03 %)	918 (20.75 %)	503 (6.05 %)	<.0001	<.0001	0.010
Hyperlipidemia	177 (44.12 %)	2051 (57.01 %)	2286 (32.50 %)	<.0001	<.0001	<.0001
High blood pressure	119 (29.46 %)	2020 (58.99 %)	2351 (38.35 %)	0.009	<.0001	<.0001
Insulin resistance	81 (47.34 %)	2147 (66.03 %)	442 (12.24 %)	<.0001	<.0001	<.0001
Heart disease	47 (9.46 %)	540 (13.83 %)	558 (6.79 %)	0.119	<.0001	0.040
Asthma	44 (14.62 %)	394 (12.69 %)	433 (7.87 %)	0.001	<.0001	0.468
NHANES cycle,				0.135	0.696	0.152
2001–2002	71 (24.07 %)	541 (16.24 %)	1022 (16.73 %)			
2003–2004	49 (16.81 %)	534 (17.15 %)	856 (15.29 %)			
2005–2006	41 (13.80 %)	499 (16.98 %)	893 (16.47 %)			
2007–2008	75 (18.37 %)	630 (17.07 %)	1056 (16.63 %)			
2009–2010	53 (11.37 %)	602 (15.22 %)	1117 (16.74 %)			
2011–2012	57 (15.58 %)	527 (17.35 %)	1038 (18.13 %)			

*P*values were reported by chi-square test for categorical variables and t-test for numerical variables;

^a^Comparisons between HCV and Control

^b^Comparisons between NAFLD and Control

^c^Comparisons between HCV and NAFLD

#### Health related quality of life in NAFLD

##### Component 1 of HRQOL questionnaire

**Fig. 2:**
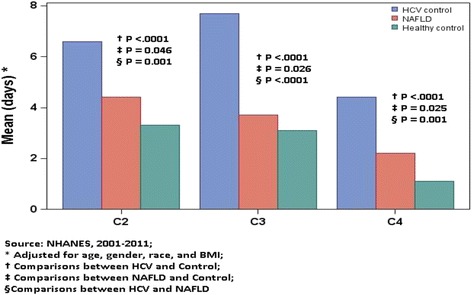
Adjusted* mean HRQOL components (C2-C4) by liver disease, and pair-wise comparisons (t-test), NHANES, 2001–2011

Component 1 of HRQOL was concerned with overall HRQOL assessment (Table 2, Fig. 2). The proportion of participants who rated their health as excellent or very good was significantly higher in the Control group as compared to both NAFLD and HCV groups (90 vs 78, 68 %, respectively; *p* < .0001). The overall HRQOL score was lowest for HCV and then NAFLD patients.

**Table 2:** HRQOL in Participants with NAFLD and HCV, NHANES, 2001–2012

Variables	HCV	NAFLD	Control	*P* value^a^	*P* value^b^	*P* value^c^
	(*n* = 346)	(*n* = 3333)	(*n* = 5982)			
HRQOL-4 components,						
C1,				<.0001	<.0001	0.005
Excellent	20 (7.19 %)	201 (6.49 %)	863 (17.36 %)			
Very good/Good	201 (60.75 %)	2112 (71.02 %)	4215 (72.22 %)			
Fair	98 (24.81 %)	831 (18.83 %)	806 (9.09 %)			
Poor	27 (7.24 %)	189 (3.66 %)	98 (1.32 %)			
C2 (no day = 0)	187 (52.47 %)	2000 (61.34 %)	4048 (68.31 %)	<.0001	<.0001	0.012
C3 (no day = 0)	175 (46.58 %)	2174 (64.07 %)	3868 (62.60 %)	<.0001	0.257	<.0001
C4 (no day = 0)	230 (64.96 %)	2663 (79.84 %)	5050 (84.41 %)	<.0001	<.0001	<.0001

*P* values were reported by chi-square test;

^a^Comparisons between HCV and Control

^b^Comparisons between NAFLD and Control

^c^Comparisons between HCV and NAFLD

##### Component 2 of HRQOL questionnaire

Component 2 was mostly involved with number of days with physical health issues. In fact, the control group was more likely to report no days of having physical health problems compared to the NAFLD group (68.3 vs 61.3 %, *p* < .001). On the other hand, HCV patients were least likely to report no days of having physical health problems (52.4 %).

##### Component 3 of HRQOL questionnaire

Component 3 was concerned with number of days with mental health issues. In this context, NAFLD patients reported similar mental health issues to the controls (62.6 vs 64 %, p > 0.05). On the other hand, HCV patients were less likely to report no days of having mental health problem, compared to controls and NAFLD patients (46.5 vs 62.6 and 64 %, *p* < 0.01).

##### Component 4 of HRQOL questionnaire

Component 4 was concerned with number of days that the physical and mental health issues would prevent patients from their activities. Again, the control group had significantly fewer number of days that physical or mental health kept the participants away from doing their usual activities (self-care, work, or recreation) during last 30-days compared to the NAFLD group (84.4 % in the Control group reported zero effected days vs. 79.8 % in NAFLD; *p* < .0001). HCV patients had the worst score with only 64.9 % in this group reporting zero effected day (*p* < .0001).

After adjusting for age, gender, race, and BMI, the association between PRO impairment and the diagnosis of HCV (C1–C4) and NAFLD (C1, C2 and C4) remained significant (Table 3).

**Table 3:** Adjusted* odds ratios (OR) with 95 % confidence intervals (CIs) for each outcome of HRQOL, NHANES, 2001-2012 (reference for C1 = excellent/very good/good and for C2–C4 = no days)

	C1	C2	C3	C4
Variable	OR ^a^ (95 % CI)			
Control	Reference	Reference	Reference	Reference
HCV	2.07 (1.48–2.88)	1.82 (1.36–2.43)	1.98 (1.52–2.58)	2.83 (2.05–3.90)
NAFLD	1.22 (1.03–1.44)	1.18 (1.03–2.08)	0.95 (0.82–1.10)	1.20 (1.02–1.41)

^a^ Adjusted for age, gender, race, BMI, education, income, smoking status, diabetes, and heart disease

(Figure 2: Adjusted* mean HRQOL components (C2–C4) by liver disease, and pair-wise comparisons (t-test), NHANES, 2001–2011).

## Discussion

Patient reported outcomes (PROs) such as HRQOL are surrogates for a patient’s experience [11]. Assessing PROs are important to accurately estimate the burden of chronic liver disease and its treatment on patients’ well-being. In this study, we used population-based data to assess HRQOL in patients with NAFLD. Our data analysis showed that NAFLD patients indeed experienced significant impairment of their HRQOL. In this context, almost one fourth (22 %) of NAFLD patients reported their health as being poor or fair which was significantly more than the healthy controls (10 %). Furthermore, this impairment resulted in a reduction of patients’ ability to perform their daily activities. Interestingly, NAFLD patients had more impairment of their physical health than their mental health. These data are partially consistent with the data previously reported showing NAFLD patients have PRO burden related to bodily pain, shortness of breath and muscle cramps as well as anxiety, unhappiness, being irritable and having mood swings [12]. Furthermore, our data is consistent with HRQOL assessment using Short Form-36 questionnaire reporting impairment in physical functioning (how much physical activities are limited), role physical (how much physical health impacts work and daily activities), bodily pain (limitations because of pain), vitality (how tired/full of energy subject feels), and role emotional (the impact of emotional problems on work and daily activities) [13].

Although not exactly clear, the reported poor physical health in NAFLD patients may be related to fatigue. Fatigue has been shown in previous studies to be a significant problem for NAFLD patients [8, 14–16]. In fact, in one such study, autonomic dysfunction and fatigue were both common in NAFLD [14, 17]. This could provide some mechanistic pathway for the development of fatigue in patients with NAFLD.

It is also important to note that impairment of HRQOL in NAFLD was less pronounced than those with HCV. In fact, the mental health aspect of PROs was more profoundly affected in HCV than NAFLD. This is not a surprise given the strong association of HRQOL with depression in patients with HCV [18–20].

## Conclusion

In conclusion, NAFLD is associated with impairment of patients’ HRQOL. Since previous studies reporting association of NAFLD with PRO impairment (12–13) were reported from the tertiary care centers, their results could have been associated with referral bias. This current study using large population database from NHANES provides additional data documenting impairment of HRQOL in patients with NAFLD. This integrated approach to understanding the clinical and PRO burden of NAFLD can provide a more comprehensive approach to treatment and management of patients with NAFLD.

### Ethics approval

This study was approved by the IRB, the approval number is NHANES IRB: 12.1074.

### Availability of data

In this study, NHANES data was used, which is available online at NHANES website.

## Appendix

**Table 4:** Spearman rho correlation for rating* their health status as poor to excellent (C1 component) with demographic variables, stratified health conditions, NHANES, 2001–2012

	rho , P				
Variables	Total (overall),	HCV	NAFLD	Heart disease	Diabetes
	*n* = 9661	*n* = 346	*n* = 3333	*n* = 1145	*n* = 1477
Age (years)	0.14, <0.01	0.11, 0.03	0.10, <0.01	−0.12, <0.01	−0.06, 0.06
Body Mass Index (BMI) (kg/m^2^)	0.23, <0.01	0.16, 0.04	0.12, <0.01	0.16, <0.01	0.13, <0.01
Gender (1 = male and 0 = female)	−0.06, <0.01	−0.11, 0.08	−0.16, <0.01	−0.12, <0.01	−0.13, <0.01
White (1 = yes and 0 = no)	−0.19, <0.01	−0.10, 0.09	−0.16, <0.01	−0.20, <0.01	−0.14, <0.01
Black (1 = yes and 0 = no)	0.06, <0.01	0.08, 0.30	−0.01, 0.44	0.11, <0.01	0.02, <0.01
Other (1 = yes and 0 = no)	0.15, <0.01	0.04, 0.43	0.18, <0.01	0.13, <0.01	0.13, <0.01
C2: days physical health, which includes physical illness and injury not good	0.30, <0.01	0.45, <0.01	0.34, <0.01	0.41, <0.01	0.39, <0.01
C3: days mental health, which includes stress, depression, and problems with emotions not good	0.17, <0.01	0.30, <0.01	0.21, <0.01	0.28, <0.01	0.26, <0.01
C4: days poor physical or mental health keep him/her from doing your usual activities, such as self-care, work, or recreation not good	0.21, <0.01	0.39, <0.01	0.24, <0.01	0.28, <0.01	0.27, <0.01

*Ranges from 1 to 5 with a higher score indicates worse self-health assessment (1 = excellent; 2 = very good; 3 = good; 4 = fair; and 5 = poor)

## Abbreviations

ALD: alcoholic liver disease
BMI: body mass index
CDC: center for disease control and prevention
CH-C: chronic Hepatitis C
CLD: chronic liver disease
FLI: fatty liver index
HRQOL: health related quality of life
NAFLD: nonalcoholic fatty liver disease
NHANES: National Health and Nutrition Examination Survey
PRO: patient reported outcome
